# SPRINT: A new parallel framework for R

**DOI:** 10.1186/1471-2105-9-558

**Published:** 2008-12-29

**Authors:** Jon Hill, Matthew Hambley, Thorsten Forster, Muriel Mewissen, Terence M Sloan, Florian Scharinger, Arthur Trew, Peter Ghazal

**Affiliations:** 1 EPCC, The University of Edinburgh, James Clerk Maxwell Building, Mayfield Road, Edinburgh, EH9 3JZ, UK; 2 Division of Pathway Medicine (DPM), The University of Edinburgh Medical School, Chancellor's building, 49 Little France Crescent, Edinburgh, EH16 4SB, UK

## Abstract

**Background:**

Microarray analysis allows the simultaneous measurement of thousands to millions of genes or sequences across tens to thousands of different samples. The analysis of the resulting data tests the limits of existing bioinformatics computing infrastructure. A solution to this issue is to use High Performance Computing (HPC) systems, which contain many processors and more memory than desktop computer systems. Many biostatisticians use R to process the data gleaned from microarray analysis and there is even a dedicated group of packages, Bioconductor, for this purpose. However, to exploit HPC systems, R must be able to utilise the multiple processors available on these systems. There are existing modules that enable R to use multiple processors, but these are either difficult to use for the HPC novice or cannot be used to solve certain classes of problems. A method of exploiting HPC systems, using R, but without recourse to mastering parallel programming paradigms is therefore necessary to analyse genomic data to its fullest.

**Results:**

We have designed and built a prototype framework that allows the addition of parallelised functions to R to enable the easy exploitation of HPC systems. The Simple Parallel R INTerface (SPRINT) is a wrapper around such parallelised functions. Their use requires very little modification to existing sequential R scripts and no expertise in parallel computing. As an example we created a function that carries out the computation of a pairwise calculated correlation matrix. This performs well with SPRINT. When executed using SPRINT on an HPC resource of eight processors this computation reduces by more than three times the time R takes to complete it on one processor.

**Conclusion:**

SPRINT allows the biostatistician to concentrate on the research problems rather than the computation, while still allowing exploitation of HPC systems. It is easy to use and with further development will become more useful as more functions are added to the framework.

## Background

### Definition of Problem

The last few years have seen the widespread introduction of high-throughput and highly parallel post genomic experiments to biological research, leading to hardware bottlenecks in the analysis of such high-dimensional data. Microarray-based techniques are a prominent example, allowing for simultaneous measurement of thousands to millions of genes or sequences across tens to thousands of different samples [1]. These measurements can represent the expression of all genes in the human genome across thousands of cancer tissue samples, or the individual gene sequence differences between thousands of patients [2,3]. These studies have generated an unprecedented amount of data and tested the limits of existing bioinformatics computing infrastructure, for example, whole genome analysis becomes intractable for any experiment with more than a few hundred arrays, depending on hardware available. Emerging whole genome associative studies and clinical projects will require from several hundreds to several thousands of microarray experiments. The complexity increases even further when considering the meta-analysis of combined data from several experiments. Microarray experiment repositories such as ArrayExpress [4] are constantly growing in size and this trend is set to continue as advances in technology are constantly contributing to an increase in the amount of data to be analysed. Increase in coverage allows for more gene sequences to be analysed on one single array. The reducing cost of this technology has also fuelled its popularity. As a consequence even larger amounts of data are being produced. The analysis of such data has become intractable on all but the most powerful hardware which often implicitly requires specialist knowledge of parallel programming.

**Figure 1:**
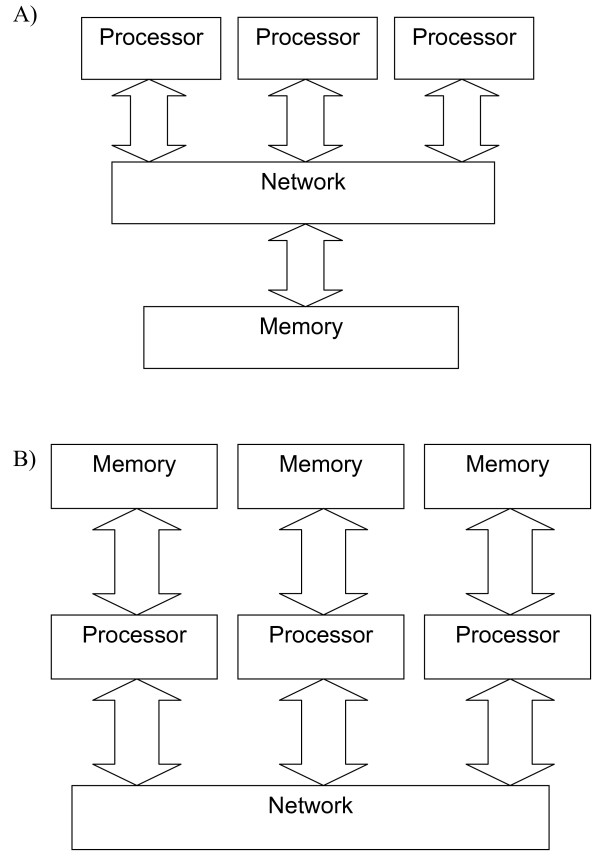
**Parallel architectures**. A) A block diagram of a shared memory system in which all processors access the same memory by means of a network. B) A block diagram of a message passing system in which memory is private to a processor. Processors share data by explicit communication over the network.

Many biostatisticians use R to process the data gleaned from microarray analysis [5-9] and there is even a dedicated group of packages, Bioconductor, for this purpose [10]. In recent years, in an attempt to address the problem of large-scale analysis, some groups in the R open source community have contributed packages that enable R code to run on multiprocessor or cluster platforms. These packages fall into two major groups: parallel "building blocks" and "task farm" packages. The first group provide fundamental parallel building blocks such that a parallel implementation of an algorithm can be constructed. The data and algorithm can be arbitrarily complex with data being passed between processors to accomplish the necessary task. However, to effectively utilise the additional processing power that this approach brings, one must have knowledge of parallel programming and perform substantial modifications to any existing R scripts. These "building blocks" are based around standard HPC programming libraries, compilers and other tools, the most popular of which are OpenMP [11] and MPI (Message Passing Interface) [12]. These two programming interfaces match onto common High Performance Computing (HPC) hardware – the shared memory system (Figure 1A) and the distributed memory system (Figure 1B). However, MPI can also be run on a shared memory system as the actual implementation of communication is independent of the functionality.

**Figure 2:**
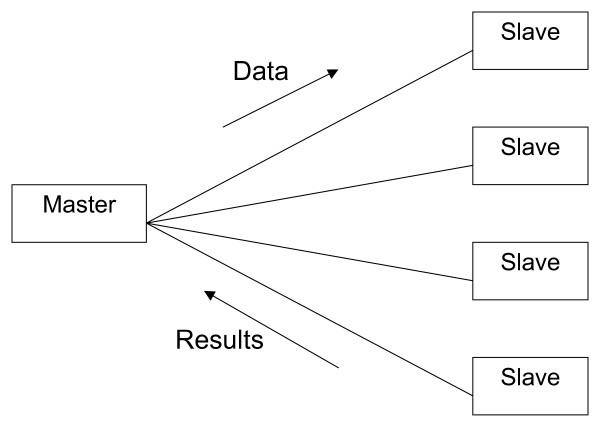
**A task farm in which data is sent from the master to the slave processes**. The slaves process the data and return a result. The slaves do not communicate with each other, only the master.

In contrast, the second group implement a task farm approach, where a 'master' process feeds a pool of 'slave' processes with jobs (Figure 2). The jobs on the slave processors are independent in that they do not communicate with other slaves. Therefore, typical uses are performing the same algorithm on each slave, but work on different data; or perform different analyses on the same data. Little knowledge of parallel processing is required here, but substantial changes to an existing R script must still be carried out.

A brief overview of eight such packages is presented here bearing in mind their applicability to analysing large genomic datasets by a statistician (that is not an expert in parallel programming). Firstly, we present the packages that can be used to create bespoke parallel functions within R. We then examine the second category of parallelisation – the task farm.

### Parallel Building Block Packages

The technologies in this group allow a programmer to distribute data and work across processors when the data is not independent to the processor that holds the data and therefore communication between processors is necessary. They therefore require knowledge of parallel programming to make full use of them, but are flexible. All of the following packages are available from the Comprehensive R Archives Network (CRAN) [13].

#### NWS and Sleigh

NWS (NetWorkSpaces) implements a shared memory system. It allows machines in a cluster to share specified variables, i.e. the same variable, with the same value, exists on all machines in the cluster. Sleigh is built on top of NWS and included in the same package and provides tools for task farming.

#### Rmpi

Rmpi is a simple wrapper around the MPI library that offers an R function for most MPI functions. MPI is the recognised standard for communication among processes that model a parallel program running on a distributed memory system. This wrapper offers very low level parallelism but requires programmers to have an extensive knowledge of parallel programming and requires significant alterations to existing scripts.

#### Rpvm

Rpvm is similar to Rmpi and provides a wrapper around the Parallel Virtual Machine (PVM) message passing library [14]. The PVM library has been almost completely superseded by MPI.

### Task Farm Packages

The packages in this section allow data to be processed in parallel provided that it is independent of the processor; therefore there is no need for communication between processors during execution. For example, performing the same analysis on many datasets could be done in parallel, as could examining independent subsets of data with the same algorithm. Many of the packages described below have overlapping functionality. All of the following packages are available from the Comprehensive R Archives Network (CRAN) [13].

#### Biopara

This package allows the execution of code fragments via secure shell on remote systems. This solution is unsuitable for typical HPC platforms because it is not usually possible to access the internal nodes of a HPC cluster via SSH where advanced knowledge of the nodes to be used is required. It also requires invasive code modifications and up-front knowledge of which machines are going to form the compute cluster.

#### R/Parallel

This package is capable of parallelising loops using minimal editing of existing R scripts [15]. This parallel R solution is of particular use in studies which involve permutation tests or heuristic searches [15], but cannot be used to solve problems where the data is dependent on other loop iterations. It is implemented using threads in C++ and as such, in the first stage at least, is only capable on running on shared memory systems.

#### Papply

This package implements a parallel version of the R command 'apply' which applies a specified expression to every element of a list. Parallelism is achieved with a task farm. There can be no dependence between list elements as no guarantee can be made regarding execution order. The user is responsible for splitting the data into chunks, one per processor used.

#### Simple Network Of Workstations (SNOW)

Snow provides a task farm Application Programming Interface (API) which allows for a single expression to be carried out on subsets of data. Snow is low level and so the user must understand the technicalities of parallel computing but it does provide greater control to the programmer. It does, however, require more extensive and non-trivial modifications to the original R program.

#### TaskpR (Task-Parallel R)

This package works by creating and managing a task farm. It is possible to pass an expression to the master and have it distributed in some non-specified manner between the slaves. Modifications to the original program are needed such as identifying which expressions are the most resource hungry and therefore will benefit most from farming out.

The parallel building block category of packages can be used to solve non-trivial problems in parallel, such as the calculation of a correlation matrix, but the user requires significant understanding of parallel programming, software and hardware architecture. Such knowledge is not common within the biological and statistical research community.

The task farm category of packages only addresses problems that benefit from simple parallelism. They are well suited for tasks such as analysing the same data with different analysis model parameters or running the same analysis on different data. However, any change in the type of analysis or the analysis method may require significant renewed customisation of R code.

As an example of the type of problem that might be faced by biostatisticians – the correlation between all values in the input data – it is helpful to look at the above packages are consider which might be used. This more computationally complex problem can be solved on HPC resources using the first set of R parallel packages, but cannot be solved on HPC resources using the second category of task farms. A solution to this and other similar problems encountered in the statistical analysis of post genomic biological data is to create such an easy-to-use interface to parallel versions of the commonly used analytical routines such as correlation and other similarity metrics. Such a tool will both increase the speed and size of data that biostatisticians can be analyse easily.

## The SPRINT framework

**Figure 3:**
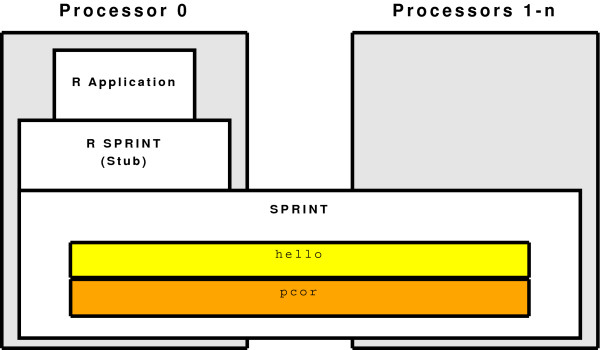
**The SPRINT framework**. SPRINT runs across all processors. The R application (which runs the R script) runs on the master processor. This links to the SPRINT library via the R to C interface. The compute farm library uses files to communicate with compute farm which can then execute functions in the library over this compute farm. The pcor library is a parallel function library (in this case parallel correlation). Other libraries can be added, such as "hello" which is a simple "Hello World" function.

In order to enable biostatisticians easy access to HPC platforms, we have created the Simple Parallel R INTerface (SPRINT) framework. SPRINT is essentially an R wrapper to parallelised statistical algorithms. The design of SPRINT uses the functionality that exists in R to transfer data to another executable process that can be written in C or FORTRAN – common languages on HPC platforms. In essence, SPRINT is a "compute farm"; it manages a number of processors that can be used for any purpose. As shown in Figure 3, the R executable and the R script used to carry out the analysis is only run on one of these processors, while SPRINT runs on all processors (including the one that R uses). When the R script encounters a function that has a parallel implementation in SPRINT, for example cor which has a parallel replacement called pcor, R then passes the necessary data to SPRINT, which carries out the correct function in parallel and passes the data back to R (Figure 3).

Given the problem of running analysis on a large dataset, a typical solution for a HPC platform would be to write code using either C or FORTRAN and implement parallelism using either OpenMP or MPI. This requires creating not only the algorithm itself, but the user-interface and I/O routines. For a small example like this, these are rarely major issues, but if any software is to be widely used by a community it must use a standard interface that is suitable for that community. As the biostatistics community uses R heavily, this means using the R interface would be an advantage. With this in mind, one could use existing R modules to write a parallel version of the R cor function, for example, with Rmpi being the obvious choice of existing R packages to use. However, a biostatistician would need to learn the parallel programming paradigms necessary and a HPC programmer would need to learn R. SPRINT bypasses both of these issue by allowing the HPC programmer to use whichever tool is most suitable for the problem in hand and the biostatistician to continue to use R as normal. It must also be considered that C and FORTRAN usually produce faster programs as they are not interpreted as R is, although this may not always be the case (as indeed R is written in C at the base level). In addition, in order to be widely adopted such a solution would need to be easy to use and be flexible in terms of hardware support.

SPRINT is written in C and uses the MPI library for communication between processes. This combination of language and parallel library is available on a wide range of platforms and generally gives very good performance. MPI uses the message passing paradigm where data is local to a particular processor and in order to share it with another processor a message containing the data must be sent to the other processor. Its use is more widespread than other parallelisation techniques, such as threading, as it will work well on almost any multi-processor platforms, from a dual-core machine to Massively Parallel Processing (MPP) architectures, such as BlueGene/P [16], though it is explicitly designed to run on typical HPC platforms. MPI programs can arbitrarily split themselves into groups, known as communicators. Most programs (SPRINT included) use only the main communicator, MPI_COMM_WORLD, which spans all processors. As such, we rely on only one feature of the MPI2 standard [12] using the MPI launch program (mpiexec) to launch two executables that share the same MPI_COMM_WORLD. This enables us to launch R on a single processor, while using the rest of the available processors only to launch SPRINT. When a parallel processing command is reached within R, all processors can then participate in the algorithm.

An R script, that uses one of the functions contained in the SPRINT framework, will go through the following stages:

• Job script requests n processors: 1 for R and SPRINT, n-1 for SPRINT only. The job is submitted to the back-end of the HPC system

• R is launched on a single processor (the master processor) and SPRINT is launched on all processors (via mpiexec, see later for details)

• R script includes SPRINT R library stub, which interfaces with SPRINT

• R script calls a parallel function contained in SPRINT

○ The stub in R tells SPRINT which function to execute

○ The stub for that function sends data to SPRINT

○ All processors call the correct parallel function

○ Result collected on master processor and returned to R

• R script call the pterminate function which shuts down SPRINT

• R script ends

To launch the two applications using the same MPI_COMM_WORLD, the following mpiexec command is used:

mpiexec -n 1 R -f $SCRIPT_NAME: -n $FARM_SIZE $SPRINT_LOCATION

The shell variables $SCRIPT_NAME, $FARM_SIZE and $SPRINT_LOCATION are the R script, an automatically created variable which gives the number of processors requested minus one for SPRINT to run on and the location of the SPRINT executable, respectively. Although the SPRINT compute farm is given one less processor than is available, it can still use the master processor on which R is running.

In this paper we describe a single parallel function, implemented within the SPRINT framework: a parallelised pairwise calculated correlation matrix. With this example function we show the potential SPRINT has for producing a step change in the size of data that can be processed and analysed using R. However, SPRINT is not limited to a single function. The framework is designed to be extended and the hope is that the computationally intensive parts of R can be ported into SPRINT and parallelised.

### Parallel Correlation – a practical implementation using SPRINT

We selected a frequent analysis problem with high-dimensional biological data as a representative case to solve using our parallelisation framework. The computation of a pairwise calculated correlation matrix is part of many clustering and classification algorithms, e.g. identifying sets of genes with similar gene expression patterns across all biological conditions in a microarray study. In this example we correlate each gene with every other gene. In practical terms this equates to correlating each row of a two-dimensional array with every other row to produce a triangular matrix of correlation coefficients. This cannot be solved with a simple task-farm approach.

There are two possible approaches to solving this problem. The first is to replicate the data on all processors. This makes programming the correlation much simpler, but limits the amount of data that can be processed as the memory on each processor must be large enough to hold all the input and output data. The second approach is to distribute the data. This removes the memory limit (depending on the size of the problem and the number of processors used), but introduces much more complex data access patterns and therefore is much more difficult to program. For this test case we chose to replicate data.

The parallel correlation algorithm is quite straightforward. A master processor is used to co-ordinate the rest of the processors. Each processor takes a row to correlate with every other row of the input matrix. Clearly, one can reduce the amount of work by not repeating rows that have already been correlated against. However, this leads to a load imbalance where the first processor gets the first row and has to correlate this to all other rows, whereas the penultimate row only has to be correlated with the final row. In order to reduce the load imbalance, a "first come, first serve" basis is used to distribute rows to slave processors. The processors are given the next row as they complete their current one until no more are left.

**Figure 4:**
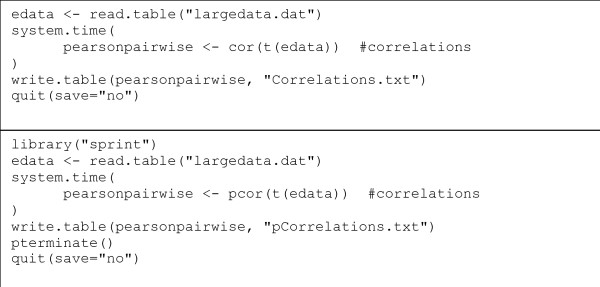
**Altering the R script**. The upper box shows the original R script used to carry out the correlation. The lower box shows the modified R script. Only two additional lines are needed and the function (cor) is changed to pcor.

The resulting R script is extremely similar to the original serial, script (Figure 4). The only changes necessary are those to include the parallel library, call the parallelised correlation and shut down the framework.

## Results

### Testing and Scaling Results

The results from our simple test show the worth of HPC in genomic analysis as well as demonstrating the usefulness of a parallel version of some R functions. Our test input data consisted of 11,000 rows (genes) of 321 columns (samples); a total of 83 Mbytes of data. The output data an 11,000 by 11,000 matrix consisting of the correlation coefficients and is 1.3 Gbytes in size. The parallel correlation function produces identical output to that of the standard ("Pearson") correlation function which mirrors the correlation coefficients along the diagonal to produce a symmetrical matrix. While a considerable amount of memory could be saved by only storing non-zero elements, the entire result would still need to fit in memory on one processor in order to pass back the correct result, in the correct format, to R. Therefore, replicating R's built-in function imposes restrictions on what is possible, but is a necessary step to ensure compatibility.

We used the Edinburgh Compute and Data Facility (ECDF) [17] to test the parallel correlation function. The ECDF consists of 1548 AMD Opteron processors, each with 2 Gbytes of memory. The system uses Sun GridEngine [18] as a batch system and jobs must be submitted to the batch system for executing. Running the test data on varying numbers of processors shows that increasing processor numbers does reduce the time taken to perform this calculation. Times were captured using R built-in system.time command.

**Figure 5:**
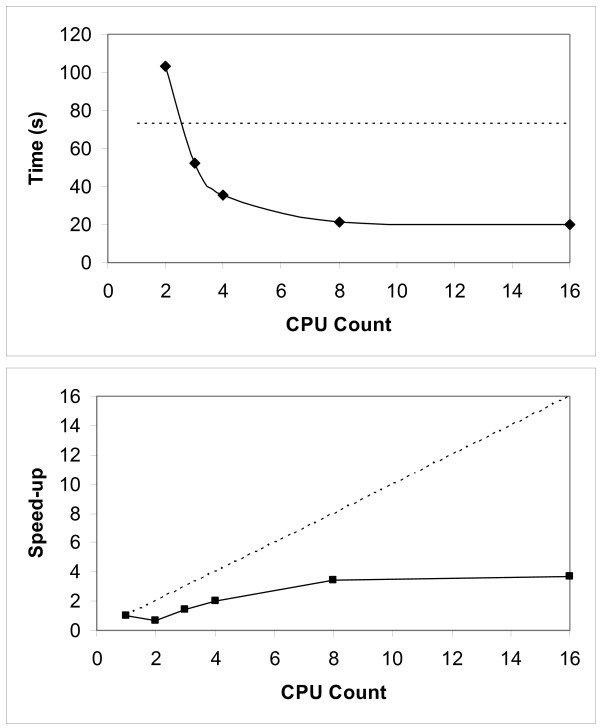
**Performance and strong scaling of the parallel correlation function**. Top graph shows the executing time of the correlation function. The dashed horizontal line is the time taken for R to execute the correlation on a single processor. The solid line shows the time for the parallelised version within SPRINT. The bottom graph shows the strong scaling (same data, different number of processors) for the parallel correlation function within SPRINT. The straight, dashed line shows linear scaling based on the execution time of R running on a single processor.

The scaling of this test function is limited as the performance increase starts to drop at around 4 processors (Figure 5). However, the executable time for the correlation is reduced from 72 seconds when using R sequentially to just 21 seconds when running the SPRINT parallel implementation over eight processors. The speed-up will increase with larger datasets. Changing the data handling strategy to a distributed one will mean that a problem that is currently intractable in serial due to memory limits will become tractable on multiple processors.

## Discussion and conclusion

In this paper we have presented a framework, SPRINT, for executing R scripts using newly created parallelised functions. We believe this framework has a widespread appeal as it requires little knowledge of parallel processing, but instead leverages on users' existing knowledge of R. Knowledge of parallelisation is only required by those wanting to add new parallel functions to the framework, not by the users of the interface. Existing R scripts need only minor modifications in order to utilise any parallelised function contained within SPRINT.

Adding new parallel functions to SPRINT is straightforward and we plan to implement more 'bottleneck' functions in the near future. By using MPI, rather than threading techniques such as OpenMP, SPRINT can be used on a wide range of systems, from a cluster of PCs to HECToR [19], the UK national supercomputer. The SPRINT framework is released under the GNU General Public Licence and is free to use [20]. In addition, the implementation of a function is separate from its interface thus allowing it to be improved without changes to the interface, and hence to changes to R scripts currently using the SPRINT framework are necessary.

The main issue regarding the framework proposed here is that functions have to be re-implemented which requires significant effort, but unlike other "out of the box" parallel R solutions we have greater freedom on how an algorithm is implemented and expands the range of algorithms that can be parallelised. However, alternatives that might provide similar potential to SPRINT are either re-writing R from the ground-up with in-built parallelism or wrap R with MPI. Both options need as much if not more effort and importantly require altering the R source code. Re-writing R with in-built parallelism would be the preferable solution as no modification of existing R scripts would be required. However, this would clearly require a vast amount of effort to achieve and in addition needs concerted and concentrated effort. In contrast, SPRINT can be built incrementally by implementing one function at a time which will allow a core set of functions to be built early and new functions can be added as required. Hence, implementations of functions are independent of each other and can be built using community effort. Implementing a framework such as SPRINT also means that solutions to problems only need to be derived once (by the community) and placed in to the framework.

In order to solve a problem such as the parallel pair-wise correlation described above a typical R user would have a few choices. Firstly, they could write their own solver using Rmpi or Rpvm. This would require learning parallel programming techniques and programming in general. Alternatively, they could try one of the task farm solutions and write a script that solves each row-row correlation on a separate processor. This is feasible, but is unlikely to be particularly efficient. SPRINT aims to solve both of these issues. The HPC programmer can concentrate on writing efficient, correct code to solve the problem in hand. The R user can concentrate on what analysis needs to be performed and does not have to consider the underlying technology.

The current function that has been implemented is a prototype that can be improved by switching to a distributed data strategy in order to remove the memory limitation. This could not be done within R alone or using any of the existing packages. In summary, SPRINT provides a very flexible framework for extending R into the realm of High Performance Computing.

## Availability and requirements

SPRINT is available from NeSCForge at: 

The software is published under GNU General Public License. The software requires Linux, R 2.5.1 or greater, C, and a version of MPI2 installed on the cluster. It has been tested using OpenMPI and LAM.

## Authors' contributions

All authors conceived the project and design. MH, JH and FS wrote the source code for SPRINT. TF, MM and PG supplied the test data. All authors drafted the manuscript, read and approved the final manuscript.
